# From Apraxia to the Individual: Rethinking Cognitive Models Through Intrinsic Differences and Recovery

**DOI:** 10.1111/ene.70244

**Published:** 2025-06-09

**Authors:** Mathieu Lesourd

**Affiliations:** ^1^ INSERM, UMR 1322 LINC Université Marie et Louis Pasteur Besançon France; ^2^ Unité de Neurologie Vasculaire (UNV) CHU de Besançon Besançon France

**Keywords:** neuroplasticity, personalized rehabilitation, praxis, sex differences

In this issue of the European Journal of Neurology, Kleineberg et al. [1] showed on a sample of 102 left brain‐damaged patients that, although men and women performed similarly in praxis tasks, voxel‐lesion‐symptom mapping revealed distinct lesion patterns. Male apraxic patients more frequently showed lesions in the left inferior frontal gyrus compared to female patients, suggesting that the neural organization of the praxis system may differ according to sex. Studying the effect of intrinsic characteristics on the implementation of mental functions in cognitive neuropsychology is not a new idea. Among them, sex may affect performance in praxis tasks, as different performance between men and women in a mechanical problem‐solving task has been shown [2]. In neuropsychology, however, sex is often discussed as a secondary variable rather than a primary focus, with research generally emphasizing the cognitive processes themselves.

Should such variables lead us to question our neurocognitive models? We may ask whether it is relevant to revise our cognitive models considering sex or other intrinsic characteristics. At the cognitive level, the question becomes: are cognitive models of praxis different for men and women? Such a claim would challenge the principle of universality, a cornerstone of cognitivism. However, it could be argued that certain intrinsic parameters—such as sex—may act as modulators of the activation of specific modules, without altering their number, nature, or the operations they perform. A similar issue can be raised regarding the brain architecture underlying praxis. The core gesture‐related network in the left hemisphere is now well established (e.g., [3]), involving the inferior parietal lobule (IPL), the lateral occipitotemporal cortex (LOTC), and the inferior frontal gyrus (IFG). It seems unlikely that the critical regions of this network differ fundamentally between individuals. However, an alternative hypothesis is that males and females may differ in the structural connectivity between the key regions of the praxis network, which could account for the observed differences in lesion‐symptom mapping and functional organization [4].

This question becomes particularly central when considering functional recovery or cognitive neuroplasticity. Much of the current research on gesture‐related functions relies on capturing a snapshot at a given time point after a stroke. However, few studies have addressed longitudinal aspects. Yet, it is established that the neural correlates of praxis deficits evolve depending on the time elapsed since the stroke [5], highlighting the role of functional recovery. While our understanding of praxis functions has significantly advanced over the past decade, particularly by deconstructing the concept of apraxia and shifting the focus from task‐based assessments to underlying cognitive processes [6], we are still unable to fully account for the parameters that determine why some patients recover from apraxia and while others do not [7]. Here, individual differences driven by intrinsic characteristics such as sex may play a significant role. Considering these variables could provide new insights into the variability of recovery trajectories and improve the way we manage apraxic patients.

From a clinical perspective, these findings suggest that two patients exhibiting similar behavioral disorders—but whose deficits are supported by distinct neurophysiological correlates—may require different rehabilitation protocols. This is especially relevant if, for one patient, the affected region belongs to a critical area of the praxis network. Taking intrinsic factors into account could help refine rehabilitation strategies, personalize interventions, and perhaps improve the chances of functional recovery.

In sum, while neurocognitive models of praxis have become increasingly sophisticated, especially in their shift toward underlying cognitive mechanisms, they may still overlook key individual differences. Recognizing the role of intrinsic factors—such as sex—not as confounding variables but as meaningful modulators could deepen our understanding of both the structure and the plasticity of the praxis system. Beyond theoretical implications, this perspective opens promising avenues for more tailored and effective clinical interventions, ultimately bridging the gap between cognitive modeling and real‐world patient care.

## Author Contributions

**Mathieu Lesourd:** writing – original draft.

## Conflicts of Interest

The author declares no conflicts of interest.

## Linked Articles

Differential lesion patterns associated with stroke‐induced apraxia in women and men, https://doi.org.10.1111/ene.70201.

## Data Availability

The author has nothing to report.
